# Meniscal anterior and posterior horn heights are associated with MRI-defined knee structural abnormalities in middle-aged and elderly patients with symptomatic knee osteoarthritis

**DOI:** 10.1186/s12891-022-05143-w

**Published:** 2022-03-08

**Authors:** Yao Liu, Guiying Du, Jun Liu

**Affiliations:** 1 Department of Radiology, The Second Xiangya Hospital, Central South University, Changsha, Hunan China; 2 Department of Radiology, Teda International Cardiovascular Hospital, Tianjin, China; 3 Clinical Research Center for Medical Imaging in Hunan Province, Changsha, China; 4 Department of Radiology Quality Control Center, Changsha, China

**Keywords:** Knee, Osteoarthritis, Meniscal height

## Abstract

**Background:**

Meniscal morphological changes are associated with knee OA. However, the correlation of meniscal height and OA-related knee structural abnormalities is still not well understood. The purpose of present study is to investigate whether and how meniscal anterior and posterior horn heights are associated with structural abnormalities in knees with symptomatic OA.

**Methods:**

Our sample consisted of 106 patients (61 female, aged 40–73 years) with symptomatic knee OA. Kellgren-Lawrence system was used for radiographic evaluation. On sagittal sequence, medial meniscal posterior horn height (MPH), lateral meniscal anterior horn height (LAH) and lateral meniscal posterior horn height (LPH) were measured on the middle slice through the medial/lateral compartment. Knee structural abnormalities were assessed using the modified whole-organ magnetic resonance imaging score (WORMS). Associations between meniscal anterior and posterior horn heights and knee structural abnormalities were assessed using linear regression analysis.

**Results:**

Higher MPH was significantly associated with higher WORMS score for medial meniscal anterior horn lesion (*P* = 0.016) but did not have a statistical association with other WORMS parameters. Increased LAH was statistically correlated with decreased WORMS scores for lateral compartmental cartilage lesions (*P* = 0.001–0.004) and lateral compartmental bone marrow edema patterns (BMEPs) (*P* = 0.021–0.027). Moreover, LPH was negatively associated with WORMS scores for lateral compartmental cartilage lesions (*P* = 0.007–0.041) and lateral compartmental BMEPs (*P* = 0.022–0.044). Additionally, higher MPH was statistically associated with lower trochlea cartilage WORMS score and higher LAH was significantly correlated with higher WORMS score for trochlea subarticular cysts.

**Conclusions:**

Changes of LAH and LPH were inversely associated with the severity of lateral compartmental cartilage lesions and BMEPs, while higher MPH was only significantly correlated with more severe medial meniscal anterior horn lesions. Besides, MPH and LAH were also significantly associated with patellofemoral structural abnormalities. The present study provided novel information for understanding the role of meniscal morphological changes in knee OA, which would be helpful in identifying and evaluating knees with or at risks for OA.

**Supplementary Information:**

The online version contains supplementary material available at 10.1186/s12891-022-05143-w.

## Background

The medial and lateral menisci are asymmetric, wedge-shaped fibrocartilaginous discs, playing an essential role in absorbing, transmitting, and distributing mechanical stress [1]. Damage to a meniscus may compromise its functions and may then increase the risks for knee osteoarthritis (OA). A large number of studies have reported that meniscal damage and malposition are associated with OA-related knee structural abnormalities such as cartilage loss, bone marrow edema patterns (BMEPs) and subchondral cysts [2–7]. Meanwhile, investigators were also becoming more concerned about meniscus geometry of OA knees. For example, a study by Jung et al. suggested that the meniscus may be hypertrophied in OA knees [8]. Moreover, using three-dimensional quantitative methods, meniscal shape and position were found to be different between individuals with and without knee OA [9–11]. However, the association of meniscal shape, in particular meniscal height, with OA-related knee structural abnormalities is still not well understood. In a prior study, we found that both medial and lateral meniscal body heights were correlated with structural abnormalities in symptomatic knee OA [12]. Given that both medial meniscal body height and posterior horn height were previously revealed to be different between knees with and without OA, whether medial meniscal posterior horn height is also associated with OA-related knee structural abnormalities has become a question to be further explored [11]. Although it is currently unknown whether there is a difference in lateral meniscal heights between knees with and without OA, however, considering the potent role of changed meniscal shape on knee OA incidence and development, further investigation into the association of medial/lateral meniscal anterior/posterior horn heights and OA-related knee structural abnormalities is important because this may help us more efficiently identify individuals with or at risks for knee OA.

Thus, the purpose of this study was to investigate whether and how meniscal anterior and posterior horn heights are associated with OA-related knee structural abnormalities in middle-aged and elderly patients with symptomatic knee OA.

## Materials and methods

### Subjects

**Fig. 1:**
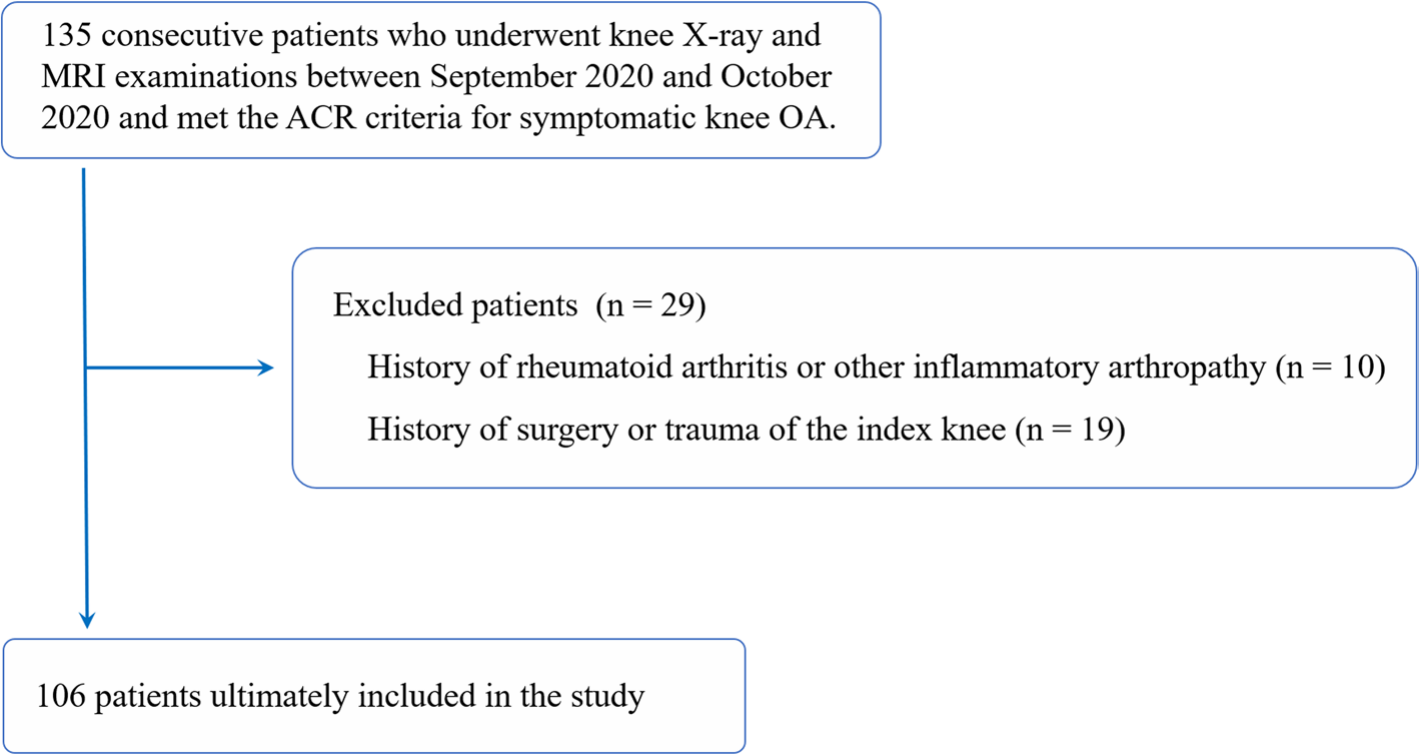
Flow chart diagram of the patient inclusion/exclusion process. OA, osteoarthritis

Our institutional review board approved this retrospective study. Informed consent was waived because the present study involved no potential risk to patients. One hundred and thirty-five consecutive patients who underwent knee X-ray and MRI examinations at the second Xiangya hospital between September 2020 and October 2020 and met the American College of Rheumatology (ACR) clinical criteria for knee OA were included [13]. In brief, patients who had knee pain and at least three of the following items were diagnosed to have symptomatic knee OA: age > 50 years; stiffness < 30 min; crepitus; bony tenderness; bony enlargement; no palpable warmth. For patients who underwent bilateral knee scans, we only included the knee presenting with more severe symptoms. The following exclusion criteria were applied: (1) history of rheumatoid arthritis or other inflammatory arthropathy; (2) history of knee surgery or trauma of the index knee. The patient selection process is described in Fig. 1.

### X-ray and MRI examinations

Weight-bearing PA using the protocol of Buckland-Wright, weight-bearing skyline and weight-bearing lateral radiographs were obtained for all kness [14, 15]. MRI scans were performed using a 3-T MRI scanner (uMR 790, Shanghai United Imaging Healthcare, Shanghai, China) with quadrature transmit-receive coils. The following sequences were used in the present study: (a) a sagittal intermediate-weighted (IW) fat-saturated (FS) 2D fast spin-echo (FSE) sequence (repetition time [TR] / echo time [TE], 2682/39 msec; in plane spatial resolution, 0.69 × 0.63 mm^2^; section thickness, 3.5 mm); (b) a sagittal T2-weighted FS 2D FSE sequence (TR/TE, 3800/68 msec; in plane spatial resolution, 0.74 × 0.63 mm^2^; section thickness, 3.5 mm); (c) a coronal IW FS 2D FSE sequence (TR/TE, 2482/38 msec; in plane spatial resolution, 0.69 × 0.56 mm^2^; section thickness, 3.5 mm); (d) a coronal T1-weighted 2D FSE sequence (TR/TE, 518/10 msec; in plane spatial resolution, 0.59 × 0.50 mm^2^; section thickness, 3.5 mm) and (e) an axial IW FS 2D FSE sequence (TR/TE, 2722/38 msec; in plane spatial resolution, 0.65 × 0.59 mm^2^; section thickness, 3.5 mm).

### Image analysis

A radiologist (Y.L. with 7 years of experience) who was blinded to subject characteristics assessed and measured all X-ray and MRI images.

#### Radiographic evaluation

Knee OA was assessed using the Kellgren-Lawrence (K&L) grades [16]. Based on this grading scheme, knee OA was divide&d into 5 grades: 0 = definite absence of X-ray changes; 1 = doubtful narrowing of joint space and possible osteophytic lipping; 2 = definite osteophytes and possible narrowing of joint space; 3 = moderate multiple osteophytes, definite narrowing of joint space, and some sclerosis and possible deformity of bone ends; and 4 = large osteophytes, marked narrowing of joint space, severe sclerosis, and definite deformity of bone ends.

#### Measurements of meniscal anterior and posterior horn heights

**Fig. 2:**
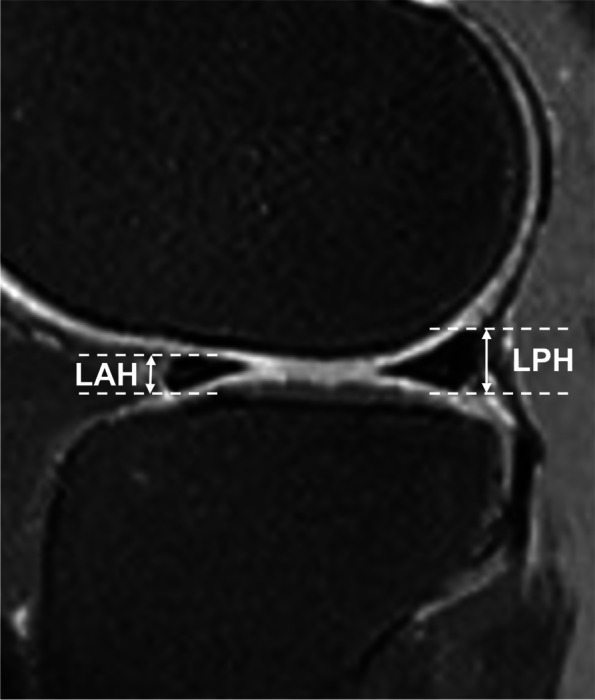
Heights of lateral meniscal anterior and posterior horn were measured on the sagittal 2D IW FS FSE sequence and were defined as the largest dimension in the longitudinal axis of the anterior and posterior horn of the lateral meniscus obtained in a middle slice through the lateral compartment. (IW, intermediate-weighted; FS, fat-saturated; FSE, fast spin-echo; LAH, lateral meniscal anterior horn height; LPH, lateral meniscal posterior horn height)

**Fig. 3:**
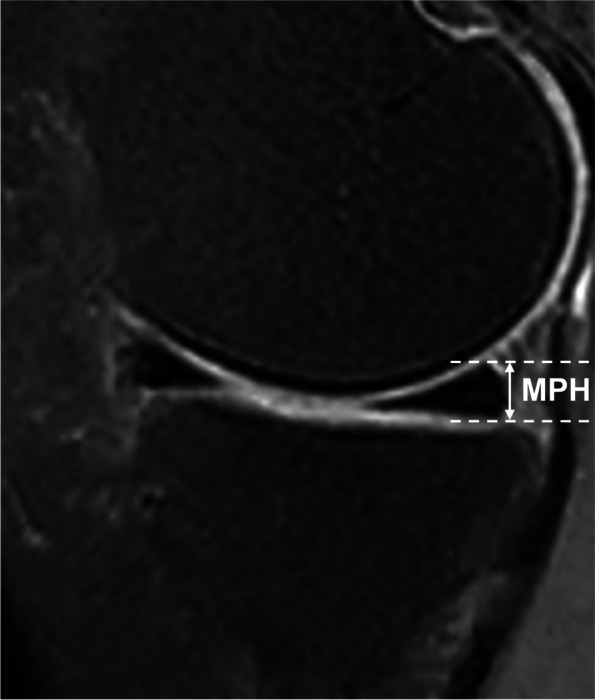
Height of medial meniscal posterior horn was measured on the sagittal 2D IW FS FSE sequence and was defined as the largest dimension in the longitudinal axis of the posterior horn of the medial meniscus obtained in a middle slice through the medial compartment. (IW, intermediate-weighted; FS, fat-saturated; FSE, fast spin-echo; MPH, Medial meniscal posterior horn height)

Medial meniscal posterior horn height (MPH), lateral meniscal anterior horn height (LAH) and lateral meniscal posterior horn height (LPH) were measured on the sagittal 2D IW FS FSE sequence. By counting the total number of slices through the medial/lateral compartment, the middle slice was selected for the measurements. Heights of the anterior and posterior horns were defined as the largest dimensions in the longitudinal axis of the anterior and posterior horns. Due to the variability in insertion patterns of the anterior horn of the medial meniscus, we were unable to find a reproducible way to measure the height of medial meniscal anterior horn [17, 18]. Hence, for the lateral meniscus we measured the heights of anterior and posterior horns, and for the medial meniscus we only measured its posterior horn (Figs. 2 and 3).

#### WORMS scoring

The University of California San Francisco (UCSF)-modified whole-organ MRI score (WORMS) system was used to semi-quantitatively assess OA-related knee morphological abnormalities [19–21]. Meniscal lesions were graded from 0 to 4 in each of 6 regions (anterior/body/posterior regions of the medial and lateral menisci) using the following 4-point scale: 0 = normal, 1 = intrasubstance signal, 2 = nondisplaced tear, 3 = displaced or complex tear, and 4 = complete destruction/maceration. Knee cartilage lesions were evaluated in medial compartment (medial femur and medial tibia), lateral compartment (lateral femur and lateral tibia), and patellofemoral compartment (patella and trochlea) with an 8-point scale: 0 = normal cartilage, 1 = normal thickness but increased or otherwise abnormal signal on fluid sensitive sequences, 2 = partial thickness focal defect < 1 cm in greatest width, 2.5 = full thickness focal defect < 1 cm in greatest width, 3 = multiple areas of grade 2 lesions intermixed with areas of normal thickness, or grade 2 lesion wider than 1 cm but < 75% of the entire region, 4 = diffuse (≥ 75% of the region) partial thickness lesion, 5 = multiple areas of grade 2.5 lesions or a full thickness lesion wider than 1 cm but < 75% of the region and 6 = diffuse (≥75% of the region) full thickness loss. BMEPs and subarticular cysts were scored from 0 to 3 in each of 6 regions (patella, trochlea, medial/ lateral femur, and medial/lateral tibia). Ligamentous/tendinous abnormalities of the anterior cruciate ligament, posterior cruciate ligament, medial collateral ligament, lateral collateral ligament, patellar tendon, and popliteal tendon as well as other findings (subchondral cysts, effusion, loose bodies, and popliteal cysts) were graded according to UCSF-modified WORMS as previously described [12, 21]. In addition, we calculated sum and maximum scores for medial and lateral meniscus individually. We also calculated sum and maximum scores for cartilage lesions, BMEPs and subarticular cysts individually for medial compartment, lateral compartment and patellofemoral compartment, respectively. The WORMS maximum score was based on the most severe lesion within all regions analyzed in the compartment.

### Reproducibility

Reproducibility results for radiographic assessment and WORMS gradings have been previously described by our group [12]. The intraclass correlation coefficients (ICCs) for intrareader agreement in K&L grades were 0.88 and 0.91, and 0.90 for interreader agreement. For WORMS gradings, ICCs range between 0.86 and 0.96 for intrareader agreement and 0.86 and 0.96 for interreader agreement.

We used ICCs to evaluate intra- and inter-reader reproducibility for meniscal anterior and posterior horn heights measurements. Ten subjects were randomly selected and two radiologists (Y.L. and G.D. with 7 and 9 years of experience, respectively) performed meniscal anterior and posterior horn heights measurements twice independently. The two readers blinded to each other’s and their own previous measurements and the two readings of each reader were at least 14 days apart. For MPH measurement, the ICCs for intrareader agreement were 0.98 and 0.96, and ICCs for interreader agreement was 0.98. The ICCs for intrareader agreement in LAH measurement were 0.95 and 0.91, and 0.96 and 0.90 for LPH measurement. The ICCs for inter-reader agreement in LAH and LPH measurements were 0.97 and 0.99, respectively.

### Statistical analysis

Statistical analyses were performed with SPSS v. 24 software (Chicago, IL), using a two-sided, 0.05 level of significance. For the variables in the characteristics of the study sample, summary statistics were constructed employing frequencies and proportions for categorical data and means and standard deviations (SDs) for continuous variables. The association between meniscal anterior/posterior horn heights and OA-related knee structural abnormalities were assessed using linear regression analysis. The analyses were adjusted for age, sex, BMI, K&L grades and were then further adjusted for meniscal anterior/posterior WORMS scores.

## Results

Our sample consisted of 106 patients (57.5% women, mean age 54.4 ± 8.8 years [range 40–73 years], mean body mass index [BMI] 24.3 ± 3.4 kg/m^2^) (Table 1). In our sample, 26 knees had K&L 0 (24.5%), while 38 knees had K&L 1 (35.8%), 33 knees had K&L 2 (31.1%), and 9 knees had K&L 3 (8.6%). During the process of radiographic evaluation, we did not meet a knee with K&L 4 grade. In addition, no knees among 106 knees were found to have lateral tibiofemoral joint space narrowing. Hence all knees with a K/L score of 3 are those with medial compartmental OA.

**Table 1:** Characteristics of the study sample

Characteristics	*n* = 106
Age, mean (SD) [range] years	54.4 (8.8) [40–73]
Female [n (%)]	61 (57.5%)
Right knees [n (%)]	58 (54.7%)
Body mass index, mean (SD) (kg/m^2^)	24.3 (3.4)
Knee Kellgren-Lawrence scores	
Grade 0 [n (%)]	26 (24.5%)
Grade 1 [n (%)]	38 (35.8%)
Grade 2 [n (%)]	33 (31.1%)
Grade 3 [n (%)]	9 (8.6%)
Medial meniscal anterior horn height, mean (SD) (mm)	5.8 (1.2)
Lateral meniscal anterior horn height, mean (SD) (mm)	4.3 (1.0)
Lateral meniscal posterior horn height, mean (SD) (mm)	6.4 (1.1)

*SD* standard deviation

Table 2 showed that higher MPH was significantly associated with higher meniscus WORMS score for MMA before and after further adjustment (*P* = 0.015 and 0.016, respectively). However, MPH was not significantly correlated with the rest medial meniscus WORMS parameters. Moreover, no statistically significant association was found of MPH with WORMS scores for medial compartmental cartilage lesions, medial compartmental BMEPs, medial compartmental subarticular cysts, ligamentous/tendinous abnormalities, effusion, loose bodies and popliteal cysts (*P* ≥ 0.060).

**Table 2:** Association between medial meniscal posterior horn height and WORMS scores for knee structural abnormalities

Outcomes	Ajusted^a^		Further adjusted^b^	
	B^c^ (95% CI)	*P* value	B^c^ (95% CI)	*P* value
Meniscal lesions				
MMA	0.09 (0.02, 0.16)	**0.015**	0.09 (0.02, 0.16)	**0.016**
MMB	0.02 (−0.12, 0.17)	0.755	0.01 (− 0.13, 0.15)	0.906
MMP	0.06 (−0.08, 0.20)	0.390		
MM sum	0.17 (−0.07, 0.42)	0.161	0.10 (−0.08, 0.28)	0.265
MM maximum	0.09 (−0.05, 0.23)	0.193	0.05 (0.32, −0.05)	0.992
Cartilage lesions				
MF	−0.08 (− 0.31, 0.15)	0.503	− 0.10 (− 0.32, 0.13)	0.409
MT	− 0.15 (− 0.35, 0.04)	0.121	−0.17 (− 0.36, 0.03)	0.090
MC sum	−0.23 (− 0.63, 0.17)	0.255	−0.26 (− 0.66, 0.14)	0.193
MC maximum	−0.09 (− 0.32, 0.14)	0.450	−0.11 (− 0.34, 0.12)	0.363
Bone marrow edema patterns				
MF	0.05 (−0.09, 0.19)	0.489	0.04 (− 0.10, 0.18)	0.578
MT	0.11 (− 0.01, 0.22)	0.074	0.09 (− 0.02, 0.20)	0.112
MC sum	0.16 (− 0.07, 0.38)	0.171	0.13 (− 0.09, 0.35)	0.239
MC maximum	0.13 (− 0.02, 0.28)	0.078	0.12 (− 0.03, 0.26)	0.108
Subarticular cysts				
MF	−0.06 (− 0.14, 0.03)	0.182	− 0.05 (− 0.14, 0.03)	0.219
MT	− 0.02 (− 0.11, 0.07)	0.707	− 0.02 (− 0.11, 0.07)	0.629
MC sum	− 0.08 (− 0.23, 0.08)	0.339	− 0.08 (− 0.23, 0.08)	0.341
MC maximum	− 0.06 (− 0.16, 0.05)	0.285	− 0.06 (− 0.17, 0.05)	0.258
Ligamentous/tendinous abnormalities				
ACL	− 0.06 (− 0.20, 0.09)	0.433	− 0.07 (− 0.21, 0.07)	0.337
PCL	− 0.08 (− 0.17, 0.01)	0.067	− 0.09 (− 0.17, 0.00)	0.060
MCL	0.04 (− 0.07, 0.14)	0.491	0.03 (− 0.08, 0.13)	0.603
LCL	0.05 (− 0.07, 0.17)	0.437	0.05 (− 0.08, 0.16)	0.463
PL	−0.06 (− 0.15, 0.03)	0.173	− 0.07 (− 0.16, 0.02)	0.126
PT	0.11 (− 0.04, 0.26)	0.162	0.11 (− 0.05, 0.27)	0.162
Effusion	−0.01 (− 0.11, 0.08)	0.806	− 0.01 (− 0.11, 0.08)	0.777
Loose bodies	− 0.03 (− 0.10, 0.04)	0.459	− 0.03 (− 0.10, 0.04)	0.428
Popliteal cysts	0.02 (−0.10, 0.14)	0.793	0.02 (−0.10, 0.14)	0.779

^a^ adjusted for age, sex, BMI and K&L grades. ^b^ further adjusted for medial meniscal posterior horn WORMS scores. ^c^ B is the regression coefficient. *CI* Confidence interval, *MMA* medial meniscal anterior horn, *MMB* medial meniscal body, *MMP* medial meniscal posterior horn, *MM* medial meniscus, *MF* medial femur, *MT* medial tibia, *MC* medial compartment, *ACL* anterior cruciate ligament, *PCL* posterior cruciate ligament, *MCL* medial collateral ligament, *LCL* lateral collateral ligament, *PL* patellar ligament, *PT* popliteal tendon

As shown in Table 3, higher LAH was significantly correlated with lower WORMS scores for LMA, LMB, LMP, LM sum and LM maximum (*P* = 0.014–0.021), while the associations were no longer significant after further adjustment (*P* ≥ 0.136). In addition, increased LAH was significantly associated with decreased lateral compartmental cartilage WORMS scores (*P* ≤ 0.031), however, after further adjustment increased LAH was only significantly associated with decreased cartilage WORMS scores for LF (*P* = 0.001) and LC sum (*P* = 0.004). Higher LAH was statistically correlated with lower BMEPs WORMS scores for LF, LT and LC sum (*P* = 0.002–0.040) and was still statistically correlated with lower BMEPs WORMS scores for LF (*P* = 0.027) and LC sum (*P* = 0.021) when fully adjusted. The correlations of LAH with WORMS scores for lateral compartmental subarticular cysts, ligamentous/tendinous abnormalities, effusion, loose bodies and popliteal cysts did not reach the statistical significance (*P* ≥ 0.119).

**Table 3:** Association between lateral meniscal anterior horn height and WORMS scores for knee structural abnormalities

Outcomes	Ajusted^a^		Further adjusted^b^	
	B^c^ (95% CI)	*P* value	B^c^ (95% CI)	*P* value
Meniscal lesions				
LMA	−0.23 (− 0.41, − 0.05)	**0.014**		
LMB	−0.20 (− 0.35, − 0.05)	**0.011**	−0.10 (− 0.24, − 0.33)	0.138
LMP	−0.14 (− 0.28, − 0.00)	**0.045**	−0.06 (− 0.19, 0.07)	0.334
LM sum	−0.57 (− 0.95, − 0.19)	**0.004**	−0.16 (− 0.38, 0.05)	0.136
LM maximum	−0.21 (− 0.39, − 0.03)	**0.021**	−0.02 (− 0.12, 0.08)	0.654
Cartilage lesions				
LF	−0.33 (− 0.49, − 0.17)	**< 0.001**	−0.27 (− 0.43, − 0.11)	**0.001**
LT	−0.32 (− 0.54, − 0.10)	**0.004**	−0.19 (− 0.39, 0.01)	0.062
LC sum	−0.65 (− 0.98, − 0.32)	**< 0.001**	−0.46 (− 0.76, − 0.15)	**0.004**
LC maximum	−0.25 (− 0.48, − 0.02)	**0.031**	−0.12 (− 0.33, 0.09)	0.267
Bone marrow edema patterns				
LF	−0.15 (− 0.26, − 0.04)	**0.010**	−0.13 (− 0.25, − 0.02)	**0.027**
LT	−0.13 (− 0.25, − 0.01)	**0.040**	− 0.06 (− 0.18, 0.05)	0.268
LC sum	− 0.28 (− 0.45, − 0.11)	**0.002**	− 0.20 (− 0.36, − 0.03)	**0.021**
LC maximum	−0.11 (− 0.26, 0.04)	0.144	− 0.06 (− 0.21, 0.09)	0.442
Subarticular cysts				
LF	−0.02 (− 0.09, 0.05)	0.567	− 0.02 (− 0.10, 0.05)	0.503
LT	0.05 (− 0.01, 0.10)	0.128	0.06 (− 0.01, 0.12)	0.068
LC sum	0.07 (− 0.05, 0.19)	0.240	0.08 (− 0.04, 0.21)	0.176
LC maximum	0.07 (− 0.05, 0.19)	0.240	0.08 (− 0.04, 0.21)	0.176
Ligamentous/tendinous abnormalities				
ACL	0.09 (− 0.07, 0.26)	0.264	0.11 (−0.07, 0.28)	0.230
PCL	−0.10 (− 0.20, 0.00)	0.054	− 0.08 (− 0.18, 0.02)	0.119
MCL	0.06 (− 0.06, 0.18)	0.315	0.07 (− 0.06, 0.19)	0.302
LCL	0.02 (− 0.12, 0.15)	0.802	0.04 (− 0.10, 0.18)	0.578
PL	0.06 (−0.04, 0.17)	0.240	0.05 (−0.15, 0.08)	0.553
PT	0.07 (−0.11, 0.24)	0.465	0.04 (− 0.14, 0.22)	0.680
Effusion	−0.03 (− 0.14, 0.08)	0.597	− 0.02 (− 0.13, 0.09)	0.710
Loose bodies	− 0.00 (− 0.08, 0.08)	0.945	− 0.00 (− 0.09, 0.08)	0.923
Popliteal cysts	0.10 (− 0.03, 0.23)	0.141	0.09 (− 0.05, 0.23)	0.208

^a^ adjusted for age, sex, BMI and K&L grades. ^b^ further adjusted for lateral meniscal anterior horn WORMS scores. ^c^ B is the regression coefficient. *CI* Confidence interval, *LMA* lateral meniscal anterior horn, *LMB* lateral meniscal body, *LMP* lateral meniscal posterior horn, *LM* lateral meniscus, *LF* lateral femur, *LT* lateral tibia, *LC* lateral compartment, *ACL* anterior cruciate ligament, *PCL* posterior cruciate ligament, *MCL* medial collateral ligament, *LCL* lateral collateral ligament, *PL* patellar ligament, *PT* popliteal tendon

Before adjusting for lateral meniscal posterior horn WORMS scores, increased LPH was statistically associated with decreased meniscus WORMS scores for LMB, LMP, and LM sum (*P* = 0.024–0.037), however, after further adjustment the statistical significances disappeared (*P* ≥ 0.289) (Table 4). Additionally, higher LPH was significantly correlated with lower scores for all cartilage WORMS parameters (*P* ≤ 0.009) and was still statistically correlated with cartilage WORMS scores for LF, LT and LC sum after further adjustment (*P* = 0.007–0.041). Similarly, increased LPH was statistically associated with decreased WORMS outcome parameters for lateral compartmental BMEPs (*P* = 0.002–0.032) before full adjustment and was still significantly associated with decreased BMEPs WORMS scores for LF (*P* = 0.044) and LC sum (*P* = 0.022) when fully adjusted. No statistically significant correlation was found between LPH and WORMS scores for lateral compartmental subarticular cysts, ligamentous/tendinous abnormalities, effusion, loose bodies and popliteal cysts (*P* ≥ 0.266).

**Table 4:** Association between lateral meniscal posterior horn height and WORMS scores for knee structural abnormalities

Outcomes	Ajusted^a^		Further adjusted^b^	
	B^c^ (95% CI)	*P* value	B^c^ (95% CI)	*P* value
Meniscal lesions				
LMA	−0.10 (− 0.27, 0.07)	0.226	− 0.01 (− 0.17, 0.14)	0.872
LMB	− 0.15 (− 0.29, − 0.01)	**0.037**	−0.07 (− 0.19, 0.06)	0.289
LMP	−0.15 (− 0.27, − 0.02)	**0.024**		
LM sum	−0.40 (− 0.76, − 0.04)	**0.029**	−0.08 (− 0.31, 0.15)	0.499
LM maximum	−0.11 (− 0.28, 0.06)	0.187	0.01 (− 0.12, 0.14)	0.892
Cartilage lesions				
LF	−0.25 (− 0.41, − 0.10)	**0.001**	−0.21 (− 0.36, − 0.05)	**0.008**
LT	−0.30 (− 0.50, − 0.10)	**0.004**	−0.19 (− 0.38, − 0.01)	**0.041**
LC sum	−0.55 (− 0.86, − 0.24)	**0.001**	−0.40 (− 0.69, − 0.11)	**0.007**
LC maximum	−0.28 (− 0.49, − 0.07)	**0.009**	−0.18 (− 0.38, 0.01)	0.068
Bone marrow edema patterns				
LF	−0.12 (− 0.22, − 0.02)	**0.025**	−0.11 (− 0.22, − 0.00)	**0.044**
LT	−0.13 (− 0.24, − 0.02)	**0.019**	−0.06 (− 0.16, 0.03)	0.201
LC sum	−0.25 (− 0.41, − 0.10)	**0.002**	−0.17 (− 0.32, − 0.03)	**0.022**
LC maximum	−0.15 (− 0.30, − 0.01)	**0.032**	−0.09 (− 0.22, 0.04)	0.170
Subarticular cysts				
LF	−0.02 (− 0.08, 0.05)	0.619	− 0.02 (− 0.09, 0.05)	0.562
LT	− 0.00 (− 0.06, 0.05)	0.925	0.01 (− 0.05, 0.07)	0.726
LC sum	− 0.04 (− 0.15, 0.07)	0.456	−0.02 (− 0.13, 0.09)	0.751
LC maximum	−0.04 (− 0.15, 0.07)	0.456	−0.02 (− 0.13, 0.09)	0.751
Ligamentous/tendinous abnormalities				
ACL	0.06 (−0.09, 0.22)	0.418	0.04 (−0.12, 0.20)	0.599
PCL	−0.04 (− 0.13, 0.06)	0.422	− 0.03 (− 0.13, 0.07)	0.547
MCL	0.07 (− 0.05, 0.18)	0.241	0.06 (− 0.05, 0.18)	0.266
LCL	0.05 (−0.07, 0.17)	0.410	0.06 (−0.07, 0.18)	0.391
PL	0.04 (−0.05, 0.14)	0.388	0.04 (−0.06, 0.14)	0.423
PT	0.01 (−0.15, 0.17)	0.917	−0.02 (− 0.18, 0.15)	0.820
Effusion	−0.02 (− 0.12, 0.08)	0.720	− 0.01 (− 0.11, 0.09)	0.832
Loose bodies	− 0.00 (− 0.07 0.07)	0.989	0.00 (− 0.07, 0.080)	0.908
Popliteal cysts	0.00 (− 0.12, 0.13)	0.960	0.02 (− 0.11, 0.14)	0.785

^a^ adjusted for age, sex, BMI and K&L grades. ^b^ further adjusted for lateral meniscal posterior horn WORMS scores. ^c^ B is the regression coefficient. *CI* Confidence interval, *LMA* lateral meniscal anterior horn, *LMB* lateral meniscal body, *LMP* lateral meniscal posterior horn, *LM* lateral meniscus, *LF* lateral femur, *LT* lateral tibia, *LC* lateral compartment, *ACL* anterior cru ciate ligament, *PCL* posterior cruciate ligament, *MCL* medial collateral ligament, *LCL* lateral collateral ligament, *PL* patellar ligament, *PT* popliteal tendon

As shown in Supplementary Table 1, higher MPH was significantly associated with lower trochlea WORMS cartilage score (*P* = 0.048). No significant association was found between MPH and other WORMS parameters for patellofemoral joint (*P* ≥ 0.209). Supplementary Table 2 showed that higher LAH was significantly correlated with higher WORMS score for trochlea subarticular cysts before and after further adjustment (*P* = 0.019 and 0.006). LAH did not have a statistical association with other WORMS parameters for patellofemoral joint (*P* ≥ 0.068). Supplementary Table 3 demonstrated that the associations between LPH and patellofemoral structural abnormalities were not statistically significant (*P* ≥ 0.058).

## Discussion

In the current study, we investigated the association of meniscal anterior and posterior horn heights with OA-related knee structural change. This study demonstrated that higher LAH and LPH were significantly associated with less severe lateral compartmental cartilage lesions and BMEPs, while increased MPH was significantly associated with more severe medial meniscal anterior horn lesions.

Morphological changes of meniscus were suggested to be one of the features of knee OA [9]. Previous studies revealed that meniscal volume, width, and height were different between patients with and without knee OA [9, 11]. A longitudinal study by Katja et al. reported that meniscal volume and width were reduced during a 2-year period in patients with medial joint space narrowing at baseline [22]. Recently, morphological changes of the meniscus were found to be related to subsequent knee replacement in knees with rapidly progressing OA [23]. The above findings indicated the important role of meniscal morphological changes in the development of knee OA. To date, the association of meniscal morphological changes and knee structural abnormalities is still not fully understood. In our previous work, we found that medial and lateral meniscal body heights were associated with multiple OA-related structural abnormalities [12]. Based on that, the present study extended the scope of the investigation to MPH, LAH and LPH, which would be a beneficial supplement in understanding the association of meniscal morphological changes and knee OA.

By comparing knees with and without radiographic OA, Wolfgang et al. found that both medial meniscal body height and posterior horn height were both higher in knees with radiographic OA [11]. Higher medial meniscal body height has already been reported to had association with meniscal lesions, cartilage lesions, BMEPs, ligamentous abnormalities and loose bodies. However, in the present study, MPH only showed a statistical correlation with medial meniscal anterior horn lesions [12]. This fact suggested that the change of MPH was less closely associated with OA-related knee structural abnormalities when compared with medial meniscal body height. A recent study by Kawahara et al. reported a statistically significant association between MPH and cartilage lesions, which conflicted with our findings [24]. A possible reason was that their results did not adjust for the common confounders such as meniscal lesions. In addition, as highly flexed knee may develop more compression on meniscus, the special lifestyle of Japanese (for example, often sit down on knees), as a special confounder, may also be a possible reason for the inconsistency of the results from two studies [25, 26].

Laterally, the changes of LAH and LPH were inversely correlated with the severity of OA-related knee structural abnormalities. A hypothetical theory raised by Wenger et al. may be helpful for explaining this finding [9]. As medial compartment of knee usually carries more loading than lateral compartment in load transmission, medial meniscus, with time, may partly or totally extrude beyond the margin of medial tibia plateau. The extruded part of medial meniscus may potentially swell as it becomes unloaded, which may cause an increase in medial meniscal height. Meanwhile, due to the narrowing of medial joint space, the relative unloaded lateral compartment is likely to develop a more open-angled shape. Lateral meniscus may therefore slightly hypertrophied and bulged, resulting in higher LAH and LPH. This hypothesis provided a possible explanation to the inverse correlation between LAH/LPH and the severity of knee structural abnormalities because higher LAH and LPH may partly resulted from the relatively decreased loading.

Interestingly, higher MPH was significantly associated with less severe trochlea cartilage lesions and higher LAH was significantly correlated with more severe trochlea subarticular cysts. Previous studies have suggested that the development of radiographic knee OA in symptomatic adults beginning in the patellofemoral compartment [27, 28]. A study by Hart et al. showed that meniscus pathology was associated with an elevated prevalence of patellofemoral cartilage lesions and was associated with worsening of patellofemoral OA-related abnormalities 2 years later [29]. However, it is not well known how meniscal height is correlated with patellofemoral OA. As mechanics plays an important role in patellofemoral pathology, further investigation on mechanical stress alteration of OA knees may be needed in exploring the association between meniscal height and patellofemoral structural abnormalities [30].

We acknowledge that this study has some limitations. First, we assessed the OA-related knee structural abnormalities using the UCSF-modified WORMS scoring system which was helpful to a detailed assessment of the knee structures [19–21]. However, as a semi-quantitative method, this scoring system may not be sensitive enough to monitor subtle changes such as cartilage compositional degeneration [21]. Hence, although in the present study meniscal anterior and posterior horn heights did not show a statistical association with some knee structural changes, quantitative study is required to further explore whether there is a potential correlation between them. Second, limited by the cross-sectional nature, this study was not able to clarify whether the changes of MPH, LAH and LPH are the causes or consequences of OA related knee structural abnormalities. A longitudinal study may contribute to further reveal the association between meniscal height and the incidence and development of knee OA and will be our future research direction. Third, our results did not adjust for meniscal anterior/posterior extrusion because no widely used measurement method and threshold has been proposed so far. Forth, we did not meet K&L 4 grade knees during the process of data collection, so our sample did not contain K&L 4 grade knees. However, since K&L 4 knees were often macerated or destroyed, a partial or total loss of meniscal substance is not uncommon in those knees. Therefore, in K&L 4 knees, the morphological changes of the meniscus may be greatly affected by the loss of meniscal substance. Regrettably, the extent of the loss of meniscal substance and how it affects the morphological changes of the meniscus were complex questions and beyond the scope of this study. Despite the above limitations, we believe this study is valuable and provides some novel information for understanding the role of meniscal morphological changes in knee OA.

## Conclusion

This study explored the association of meniscal anterior and posterior horn heights with OA-related knee structural abnormalities. We found that increased LAH and LPH were significantly associated with less severe lateral compartmental cartilage lesions and BMEPs, while MPH only had a statistical correlation with medial meniscal anterior horn lesions. Moreover, increased MPH was statistically associated with less severe trochlea cartilage score and increased LAH was significantly correlated with more severe trochlea subarticular cysts.

## Supplementary Information

**Additional file 1.**

**Additional file 2.**

**Additional file 3.**

## Data Availability

Datasets used and/or analyzed in the current study are available from the corresponding author on reasonable request.

## Abbreviations

ACL: Anterior cruciate ligament
ACR: The American College of Rheumatology
BMEPs: Bone marrow edema patterns
CI: Confidence interval
FS: Fat-saturated
FSE: Fast spin-echo
ICCs: Intraclass correlation coefficients
IW: Intermediate-weighted
K&L: Kellgren-Lawrence
LAH: Lateral meniscal anterior horn height
LC: Lateral compartment
LCL: Lateral collateral ligament
LF: Lateral femur
LM: Lateral meniscus
LMA: Lateral meniscal anterior horn
LMB: Lateral meniscal body
LMP: Lateral meniscal posterior horn
LPH: Lateral meniscal posterior horn height
LT: Lateral tibia
MC: Medial compartment
MCL: Medial collateral ligament
MF: Medial femur
MM: Medial meniscus
MT: Medial tibia
MMA: Medial meniscal anterior horn
MMB: Medial meniscal body
MPH: Medial meniscal posterior horn height
MMP: Medial meniscal posterior horn
OA: Osteoarthritis
PCL: Posterior cruciate ligament
PL: Patellar ligament
PT: Popliteal tendon
SD: Standard deviation
TR: Repetition time
TE: Echo time
WORMS: Whole-organ MRI score
