# Supplementary material for: Meniscal anterior and posterior horn heights are associated with MRI-defined knee structural abnormalities in middle-aged and elderly patients with symptomatic knee osteoarthritis
Source: BMC Musculoskelet Disord. 2022 Mar 8;23:218. doi: 10.1186/s12891-022-05143-w (PMC8903164; doi:10.1186/s12891-022-05143-w)
Supplement: Supplementary file 1 — Additional file 1. [file 12891_2022_5143_MOESM1_ESM.docx]

**Supplementary table 1.** Association between medial meniscal posterior horn height and WORMS scores for patellofemoral compartmental structural abnormalities.

| Outcomes | Ajusted^a^ | |  | Further adjusted^b^ | |
| --- | --- | --- | --- | --- | --- |
|  | B**^c^** (95% CI) | P value |  | B**^c^** (95% CI) | P value |
| Cartilage lesions |  |  |  |  |  |
| Patella | -0.04 (-0.33, 0.24) | 0.760 |  | -0.03 (-0.32, 0.26) | 0.818 |
| Trochlea | -0.27 (-0.55, 0.00) | 0.051 |  | -0.28 (-0.56, 0.00) | **0.048** |
| PTJ sum | -0.32 (-0.80, 0.17) | 0.198 |  | -0.31 (-0.81, 0.18) | 0.209 |
| PTJ maximum | -0.04 (-0.33, 0.25) | 0.806 |  | -0.03 (-0.32, 0.26) | 0.816 |
| Bone marrow edema patterns |  |  |  |  |  |
| Patella | 0.08 (-0.09, 0.24) | 0.346 |  | 0.09 (-0.08, 0.25) | 0.302 |
| Trochlea | -0.08 (-0.23, 0.08) | 0.339 |  | -0.09 (-0.25, 0.07) | 0.254 |
| PTJ sum | 0.00 (-0.24, 0.25) | 0.987 |  | -0.00 (-0.25, 0.24) | 0.974 |
| PTJ maximum | 0.03 (-0.15, 0.21) | 0.705 |  | 0.03 (-0.15, 0.21) | 0.732 |
| Subarticular cysts |  |  |  |  |  |
| Patella | -0.01 (-0.16, 0.14) | 0.028 |  | 0.01 (-0.14, 0.16) | 0.941 |
| Trochlea | -0.09 (-0.22, 0.05) | 0.196 |  | -0.09 (-0.22, 0.05) | 0.199 |
| PTJ sum | -0.09 (-0.33, 0.14) | 0.434 |  | -0.08 (-0.32, 0.15) | 0.499 |
| PTJ maximum | -0.06 (-0.23, 0.11) | 0.508 |  | -0.05 (-0.22, 0.13) | 0.597 |

**a**: adjusted for age, sex, BMI and K&L grades. **b**: further adjusted for medial meniscal posterior horn WORMS scores. **c**: B is the regression coefficient. CI: Confidence interval. PTJ: patellofemoral joint.
