# Supplementary material for: Meniscal anterior and posterior horn heights are associated with MRI-defined knee structural abnormalities in middle-aged and elderly patients with symptomatic knee osteoarthritis
Source: BMC Musculoskelet Disord. 2022 Mar 8;23:218. doi: 10.1186/s12891-022-05143-w (PMC8903164; doi:10.1186/s12891-022-05143-w)
Supplement: Supplementary file 2 — Additional file 2. [file 12891_2022_5143_MOESM2_ESM.docx]

**Supplementary table 2.** Association between lateral meniscal anterior horn height and WORMS scores for patellofemoral compartmental structural abnormalities.

| Outcomes | Ajusted^a^ | |  | Further adjusted^b^ | |
| --- | --- | --- | --- | --- | --- |
|  | B**^c^** (95% CI) | P value |  | B**^c^** (95% CI) | P value |
| Cartilage lesions |  |  |  |  |  |
| Patella | 0.16 (-0.17, 0.48) | 0.343 |  | 0.17 (-0.17, 0.51) | 0.314 |
| Trochlea | 0.22 (-0.10, 0.53) | 0.178 |  | 0.25 (-0.07, 0.58) | 0.124 |
| PTJ sum | 0.37 (-0.18, 0.92) | 0.185 |  | 0.43 (-0.15, 0.99) | 0.142 |
| PTJ maximum | 0.24 (-0.09, 0.56) | 0.151 |  | 0.25 (-0.08, 0.58) | 0.141 |
| Bone marrow edema patterns |  |  |  |  |  |
| Patella | 0.02 (-0.17, 0.21) | 0.853 |  | 0.01 (-0.19, 0.20) | 0.962 |
| Trochlea | 0.14 (-0.04, 0.32) | 0.121 |  | 0.16 (-0.03, 0.34) | 0.091 |
| PTJ sum | 0.16 (-0.12, 0.43) | 0.255 |  | 0.16 (-0.12, 0.45) | 0.258 |
| PTJ maximum | 0.13 (-0.07, 0.33) | 0.201 |  | 0.14 (-0.07, 0.35) | 0.196 |
| Subarticular cysts |  |  |  |  |  |
| Patella | 0.05 (-0.12, 0.22) | 0.566 |  | 0.04 (-0.12, 0.22) | 0.624 |
| Trochlea | 0.18 (0.03, 0.32) | **0.019** |  | 0.21 (0.06, 0.36) | **0.006** |
| PTJ sum | 0.23 (-0.04, 0.49) | 0.093 |  | 0.25 (-0.02, 0.52) | 0.068 |
| PTJ maximum | 0.15 (-0.04, 0.34) | 0.128 |  | 0.17 (-0.03, 0.37) | 0.092 |

**a**: adjusted for age, sex, BMI and K&L grades. **b**: further adjusted for medial meniscal posterior horn WORMS scores. **c**: B is the regression coefficient. CI: Confidence interval. PTJ: patellofemoral joint.
