# Supplementary material for: Meniscal anterior and posterior horn heights are associated with MRI-defined knee structural abnormalities in middle-aged and elderly patients with symptomatic knee osteoarthritis
Source: BMC Musculoskelet Disord. 2022 Mar 8;23:218. doi: 10.1186/s12891-022-05143-w (PMC8903164; doi:10.1186/s12891-022-05143-w)
Supplement: Supplementary file 3 — Additional file 3. [file 12891_2022_5143_MOESM3_ESM.docx]

**Supplementary table 3.** Association between lateral meniscal posterior horn height and WORMS scores for patellofemoral compartmental structural abnormalities.

| Outcomes | Ajusted^a^ | |  | Further adjusted^b^ | |
| --- | --- | --- | --- | --- | --- |
|  | B**^c^** (95% CI) | P value |  | B**^c^** (95% CI) | P value |
| Cartilage lesions |  |  |  |  |  |
| Patella | 0.12 (-0.18, 0.41) | 0.443 |  | 0.14 (-0.17, 0.45) | 0.377 |
| Trochlea | 0.13 (-0.16, 0.42) | 0.379 |  | 0.17 (-0.13, 0.47) | 0.263 |
| PTJ sum | 0.25 (-0.26, 0.76) | 0.342 |  | 0.31 (-0.22, 0.83) | 0.248 |
| PTJ maximum | 0.17 (-0.13, 0.46) | 0.269 |  | 0.19 (-0.12, 0.49) | 0.229 |
| Bone marrow edema patterns |  |  |  |  |  |
| Patella | 0.09 (-0.09, 0.26) | 0.319 |  | 0.10 (-0.07, 0.28) | 0.248 |
| Trochlea | 0.11 (-0.06, 0.27) | 0.206 |  | 0.13 (-0.04, 0.30) | 0.135 |
| PTJ sum | 0.19 (-0.06, 0.44) | 0.132 |  | 0.23 (-0.03, 0.49) | 0.077 |
| PTJ maximum | 0.16 (-0.03, 0.34) | 0.098 |  | 0.18 (-0.01, 0.37) | 0.061 |
| Subarticular cysts |  |  |  |  |  |
| Patella | 0.13 (-0.02, 0.29) | 0.094 |  | 0.13 (-0.03, 0.29) | 0.099 |
| Trochlea | 0.08 (-0.06, 0.21) | 0.261 |  | 0.11 (-0.03, 0.24) | 0.136 |
| PTJ sum | 0.21 (-0.03, 0.45) | 0.087 |  | 0.24 (-0.01, 0.49) | 0.058 |
| PTJ maximum | 0.15 (-0.03, 0.32) | 0.107 |  | 0.17 (-0.01, 0.35) | 0.071 |

**a**: adjusted for age, sex, BMI and K&L grades. **b**: further adjusted for medial meniscal posterior horn WORMS scores. **c**: B is the regression coefficient. CI: Confidence interval. PTJ: patellofemoral joint.
